# Prediction of Insulin Resistance by Modified Triglyceride Glucose Indices in Youth

**DOI:** 10.3390/life11040286

**Published:** 2021-03-28

**Authors:** Kyungchul Song, Goeun Park, Hye Sun Lee, Youngha Choi, Jun Suk Oh, Han Saem Choi, Junghwan Suh, Ahreum Kwon, Ho-Seong Kim, Hyun Wook Chae

**Affiliations:** 1 Department of Pediatrics, Severance Children’s Hospital, Endocrine Research Institute, Yonsei University College of Medicine, Seoul 03722, Korea; endosong@yuhs.ac (K.S.); younghachoi1986@gmail.com (Y.C.); joojang87@naver.com (J.S.O.); hansaem6890@yuhs.ac (H.S.C.); suh30507@yuhs.ac (J.S.); armea@yuhs.ac (A.K.); kimho@yuhs.ac (H.-S.K.); 2 Biostatistics Collaboration Unit, Yonsei University College of Medicine, Seoul 03722, Korea; gogoeun@yuhs.ac (G.P.); hslee1@yuhs.ac (H.S.L.)

**Keywords:** triglycerides, glucose, insulin resistance, child, adolescent

## Abstract

The triglyceride glucose (TyG) index, derived from a combination of fasting glucose and triglycerides, has been suggested as a useful marker for insulin resistance (IR), in addition to modified TyG indices that combine obesity parameters. This study investigated the association and utility of TyG and modified TyG indices for IR prediction in youth. Based on the Korea National Health and Nutritional Examination Survey, the data of 3728 youth aged 10–19 years were analyzed. Odds ratios (ORs) and 95% confidence intervals (CIs) of tertiles 2 and 3 for each parameter were calculated and compared with tertile 1 as a reference. To compare the parameters for identifying IR, receiver operating characteristic curves were plotted and the area under the curve (AUC) was calculated. The ORs and 95% CIs for insulin resistance (IR) progressively increased across tertiles of each parameter. Overall, all modified TyG indices presented higher ORs and AUC than the TyG index. The TyG-body mass index standard deviation score showed the largest AUC for IR detection in all subjects. In conclusion, TyG and modified TyG indices could be used as valuable markers for the prediction of IR in youth. Moreover, modified TyG indices had better diagnostic accuracy than the TyG index.

## 1. Introduction

Insulin resistance (IR), characterized by an inadequate physiological response with insensitivity to insulin, is a major risk factor for metabolic syndrome and cardiovascular diseases (CVD) [1,2,3]. A systematic review revealed that the prevalence of metabolic syndrome was high, at 3.3% in children and 29.2% in obese children [4]. Similarly, a population-based study in Korea found that the prevalence of metabolic syndrome had increased from 4.0% in 1998 to 7.8% in 2007 [5]. Considering the increasing prevalence of metabolic syndrome in youth and association of metabolic syndrome with risk of type 2 diabetes and CVD, it is important to detect IR in children and adolescents [6].

For measuring IR, the glucose clamp technique is considered as the gold standard [7]. However, because of its complicated and invasive nature, this test is difficult to perform in youth [8]. Therefore, homeostasis model assessment of insulin resistance (HOMA-IR) index, calculated as the product of the fasting levels of glucose and insulin, is suggested as a robust marker for IR quantification [1,9,10]. However, insulin measurement is not a routine test in the clinical setting and has standardization problems [11]. Thus, various indices combining glucose levels and lipid parameters were suggested as predictors of IR [12,13]. Among them, the triglyceride glucose (TyG) index, derived from the combination of fasting glucose and triglycerides (TG), has been suggested as a useful marker for IR in adults [14].

In addition, modified TyG indices that combine obesity indices such as body mass index (BMI), waist circumference (WC), and waist-to-height ratio (WHtR) have been suggested because obesity is closely associated with IR [15,16]. Kim et al. [16] reported that such modified TyG indices could act as alternative markers for assessing IR. Lee et al. [13] reported that the TyG index combined with BMI or WC was superior to the TyG index alone in among U.S population. However, investigations into the association between IR and the TyG and modified TyG indices are extremely limited in youth.

Therefore, this study aimed to investigate the association of TyG and modified TyG indices with IR in youth through the analysis of the Korean National Health and Nutrition Examination Survey (KNHANES) data. The objectives of our study were (1) to compare the TyG and modified TyG indices as surrogate markers for predicting IR and (2) to determine valid cut-off values of the TyG and modified TyG indices for predicting IR. 

## 2. Materials and Methods

### 2.1. Participants

**Figure 1:**
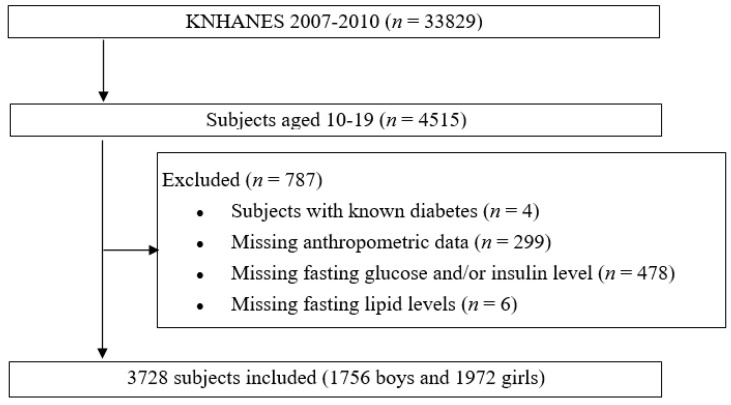
Flowchart of the study population selection process. KNHANES, Korea National Health and Nutrition Examination Survey.

This study included the data acquired in the third and fourth KNHANES, conducted from 2007 to 2010. Figure 1 depicts the flowchart of study design and patient inclusion. KNHANES is a cross-sectional and nationally representative survey with a complex, stratified, multistage probability sampling of the Korean population. It is conducted annually by the Korea Centers for Disease Control and Prevention (KCDC) based on the National Health Promotion and consists of health surveys, examinations, and nutrition surveys. These data provide a variety of information about health status and behavior, socio-economic demographics, and laboratory tests. Sample weights were used to account for differential probabilities of selection and non-response and were included in the estimation process for all analyses. The weighted data were then adjusted to represent the sex- and age-specific Korean populations [17]. KNHANES is approved by the KCDC. 

### 2.2. Study Variables

Data on age, sex, anthropometric measurement, plasma lipid levels, and insulin levels were collected. A portable stadiometer (range, 850–2060 mm; Seriter, Holtain Ltd., Crymych, UK) was used to the nearest 0.1 cm for height, and a calibrated balance beam scale (Giant 150N; HANA, Seoul, Korea) was used in the upright position to the nearest 0.1 kg for weight. BMI was calculated as weight (kg)/height squared (m^2^). The height, weight, and BMI were presented as standard deviation score (SDS) values on the basis of the 2017 Korean National Growth Charts [18]. Children were classified as normal weight (<85th percentile), overweight (85th–95th percentile), or obesity (≥95th percentile) according to their BMI. WC was measured midway between the costal margin and iliac crest at the end of a normal expiration, and WHtR was calculated as WC (cm)/height (cm). Central obesity was defined as WC >90th percentile using the Korean waist reference data [19].

### 2.3. Laboratory Analysis

Blood samples were collected from an antecubital vein after an 8-h fast, processed, and immediately refrigerated. The serum level of fasting glucose, total cholesterol (TC), high-density lipoprotein cholesterol (HDL-C), and TG were measured using the Hitachi 7600 automatic analyzer (Hitachi, Tokyo, Japan). Serum insulin was measured using the Wizard 1470 gamma counter (Perkin-Elmer, Turku, Finland).

Low-density lipoprotein cholesterol (LDL-C) was calculated using the Friedewald formula (1) [20]:

LDL-C = TC − [HDL-C + (TG/5)](1)

and non-HDL-C was calculated as TC − HDL-C [21]. The atherogenic index of plasma (AIP) was defined as log (TG/HDL-C) [22]. For quantification of IR, HOMA-IR was calculated as fasting insulin (mg/dL) × fasting glucose (mg/dL)/22.5. IR was defined as the HOMA-IR of >95th percentile for each sex and age using Korean HOMA-IR reference data [16]. TyG and modified TyG indices were defined and calculated as formula (2–6) [15,17]:

TyG index = Ln [TG (mg/dL) × fasting glucose (mg/dL)/2](2)

TyG-BMI = TyG index × BMI(3)

TyG-BMI SDS = TyG index × BMI SDS(4)

TyG-WC = TyG index × WC(5)

TyG-WHtR = TyG index × WHtR(6)

### 2.4. Statistical Analysis

The sampling weights were considered in all analyses to report representative estimates of the Korean children and adolescents. The data were analyzed using SAS, version 9.4 (SAS Inc., Cary, NC, USA), and R, version 4.0.2 (The R Foundation for Statistical Computing, Vienna, Austria; http://www.R-project.org/; accessed on 8 August 2020), for the complex survey design with clustering, stratification, and unequal weighting of the KNHANES sample. All continuous variables were expressed as weighted means with standard errors, and categorical variables were expressed as numbers and weighted percentages. The independent-samples *t*-test was used to compare continuous variables, and Pearson’s chi-square test was used to compare categorical variables. Logistic regression analyses were performed to explain the relationship between IR as the dependent variable and various markers. ORs and 95% CIs of tertiles 2 and 3 for each parameter were calculated and compared with tertile 1 as a reference. The correlation of TyG and modified TyG indices with HOMA-IR was demonstrated using a scatter plot and adjusted line. Sensitivity and specificity were calculated as the markers’ optimal cut-off values based on Youden’s index. Receiver operating characteristic (ROC) curves were plotted, and the area under the curve (AUC) was calculated to compare the relative diagnostic strengths of these parameters for identifying IR. The bootstrap method was used to perform pairwise comparisons between AUCs for the parameters. Significance was determined as *p* < 0.05.

## 3. Results

### 3.1. Baseline Characteristics of the Subjects

Table 1 shows the baseline characteristics of participants according to sex. The prevalence of IR was higher in males (13.19%) than in females (10.69%). WC, WHtR, glucose level, and proportion of subjects with central obesity or IR were higher in males than in females, while overall lipid levels were higher in females than in males. The TyG-BMI, TyG-WC, and TyG-WHtR indices were higher in males than in females, while the TyG index and TyG-BMI SDS were not significantly different between males and females.

### 3.2. ORs of TyG and Modified TyG Indices for Predicting IR

The ORs and 95% CIs for IR progressively increased across tertiles of each parameter (Table 2). All TyG and modified TyG indices exhibited significantly higher ORs and 95% CIs of tertile 3 (OR range 6.06–14.66) than that of tertile 1 (all *p* < 0.001) in the total subjects. In contrast, the lipid parameters exhibited ORs of tertile ranged as 0.43–4.25 compared with those of tertile 1 in the total subjects. Among the modified indices, TyG-WHtR presented the highest ORs and 95% CIs for IR in the total subjects (OR = 14.66) and males (OR = 21.59) while TyG-BMI SDS presented the highest ORs and 95% CIs in females (OR = 8.78). Overall, all modified TyG indices presented higher ORs and 95% CIs than TyG index and lipid profiles in the subjects.

### 3.3. Correlation of TyG and Modified TyG Indices with HOMA-IR

**Figure 2:**
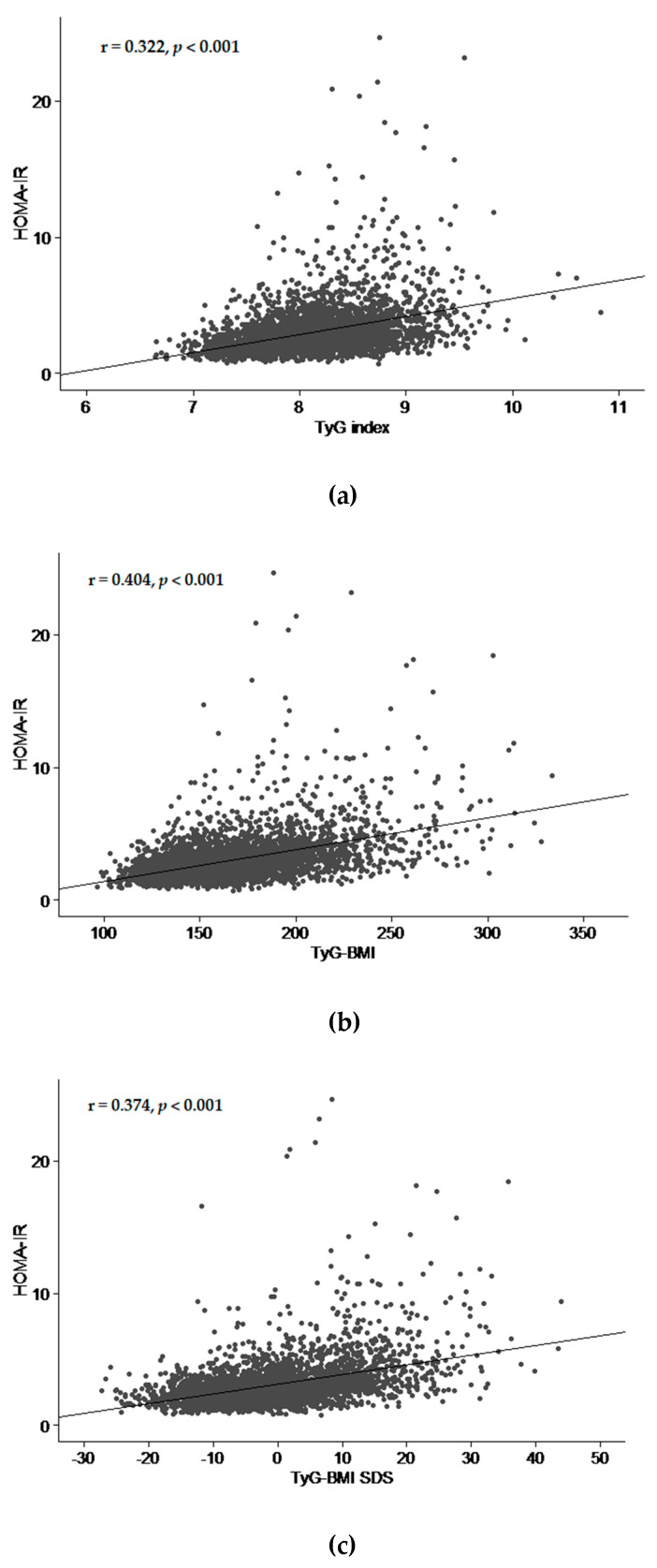

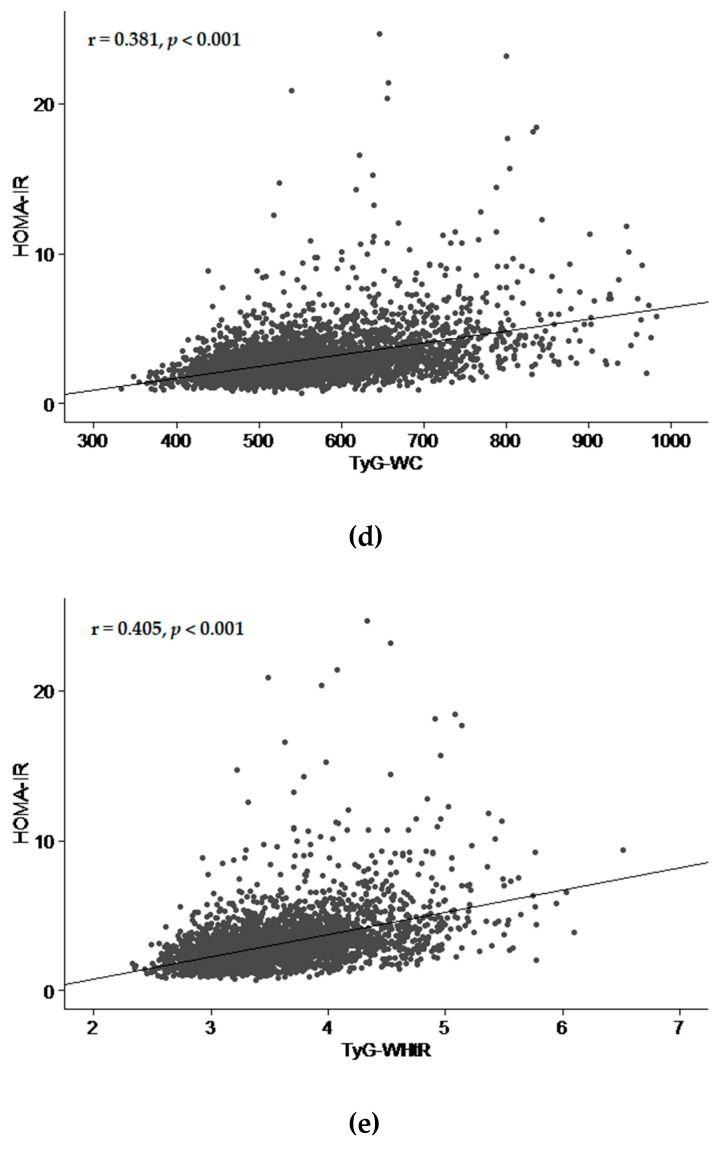
Scatter plot and fitted line of HOMA-IR and TyG index (**a**), TyG-BMI, (**b**), TyG-BMI SDS (**c**), TyG-WC (**d**), and TyG-WHtR (**e)** in all subjects. HOMA-IR, homeostasis model assessment of insulin resistance; TyG index, triglyceride glucose index; BMI, body mass index; SDS, standard deviation score; WC, waist circumference; WHtR, waist-to-height ratio.

In scatter plot and fitted line of TyG and modified TyG indices with HOMA-IR, HOMA-IR tended to increase with increasing TyG and modifed TyG indices (all *p* < 0.001) (Figure 2). Among the indices, coefficient of correlation was highest in TyG-WHtR (r = 0.405, *p* < 0.001).

### 3.4. Cut-off Values and AUC of the TyG and Modified TyG Indices for Predicting IR

**Figure 3:**
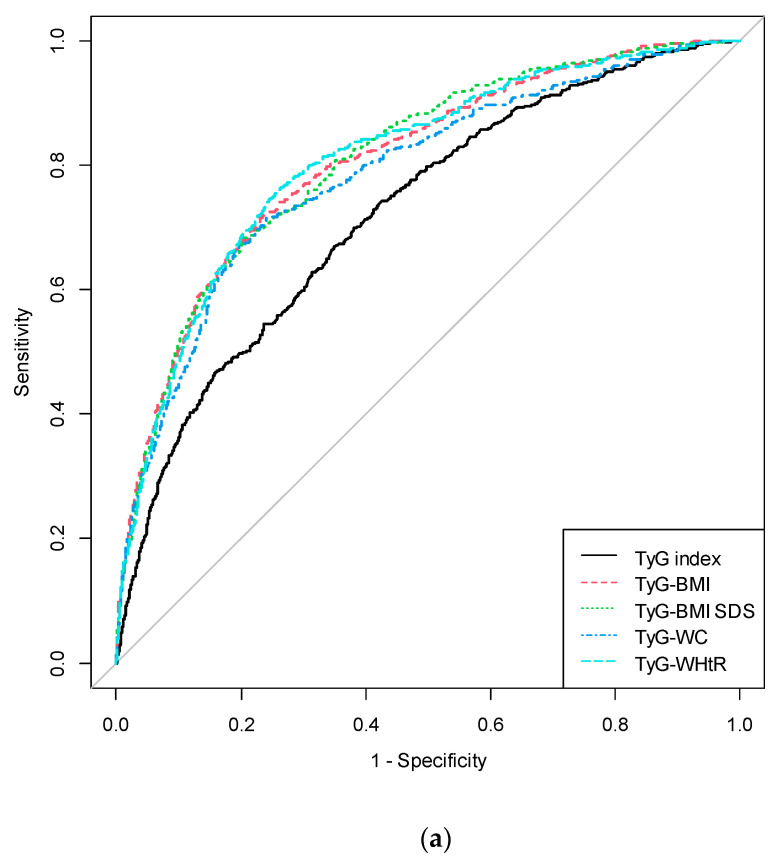

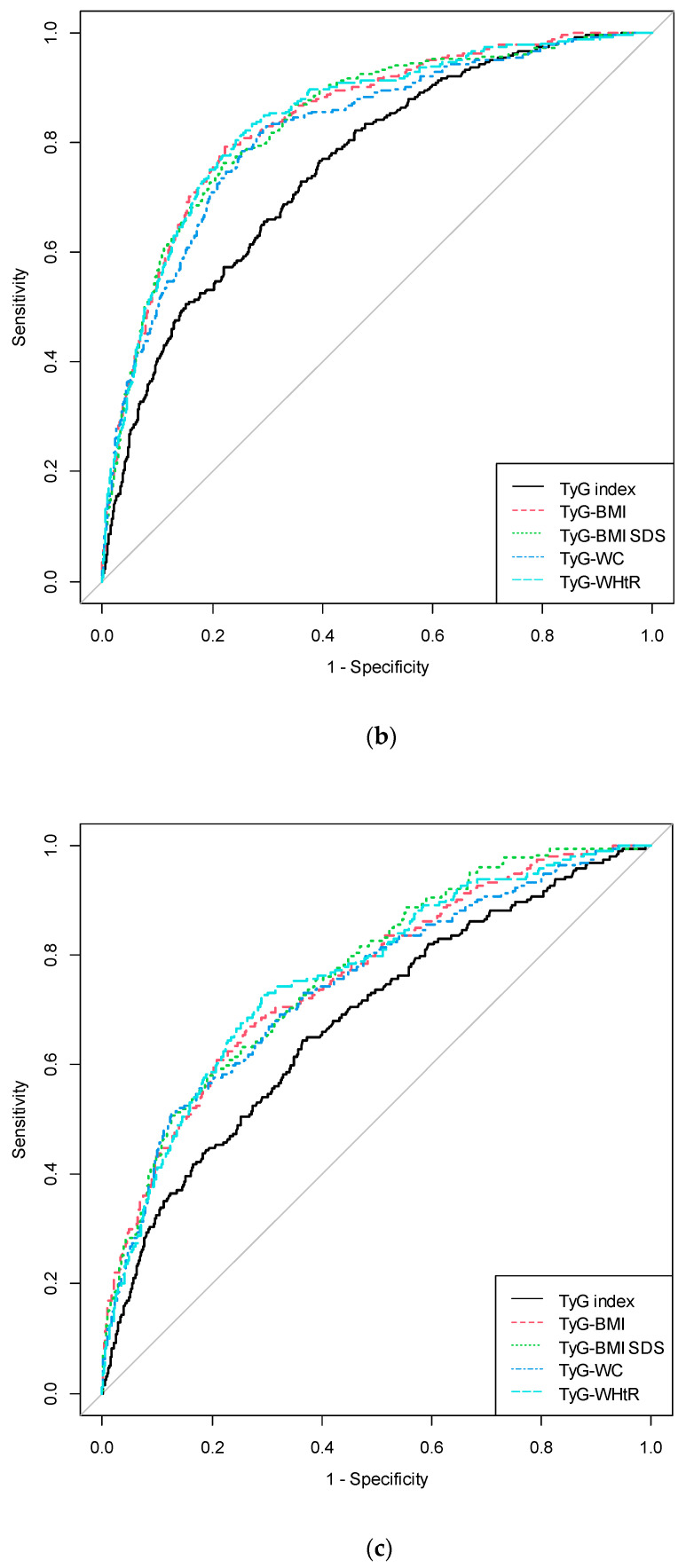
Receiver operating characteristic curve for each parameter in the prediction of insulin resistance. (**a**) ROC curve of the prediction of insulin resistance in all subjects. (**b**) ROC curve of the prediction of insulin resistance in males. (**c**) ROC curve of the prediction of insulin resistance in females. ROC: receiver operating characteristic; TyG index, triglyceride glucose index; BMI, body mass index; SDS, standard deviation score; WC, waist circumference; WHtR, waist-to-height ratio.

The results of ROC curve analyses and AUCs with the corresponding 95% CIs for TyG and modified TyG indices are shown in Table 3 and Figure 3. AUC ranged from 0.723 to 0.810 in total subjects. All TyG and modified TyG indices predicted IR significantly (all *p* < 0.001). In total subjects, the cut-off values for IR prediction were 8.261, 178.957, 5.105, 599.800, and 3.696 in TyG index, TyG-BMI, TyG-BMI SDS, TyG-WC, and TyG-WHtR, respectively (all *p* < 0.001). TyG-BMI SDS showed the largest AUC for IR detection with 0.810 in total subjects and 0.766 in females (*p* < 0.001), respectively. In males, TyG-BMI and TyG-WHtR showed the largest AUC with 0.842 for IR detection (*p* < 0.001). Overall, all modified TyG indices—TyG-BMI, TyG-BMI SDS, TyG-WC, and TyG-WHtR—presented significantly higher AUC and 95% CIs than the TyG index (Table A1). Among the modified TyG indices, TyG-BMI and TyG-BMI SDS presented significantly higher AUC and 95% CIs than TyG-WC in the total subjects. In addition, TyG-WHtR presented significantly higher AUC and 95% CIs than TyG-WC overall (total subjects, *p* < 0.001; males, *p* = 0.002; females, *p* = 0.034). 

## 4. Discussion

This study shows that the TyG and modified TyG indices can be important predictors for IR in youth. The ORs and 95% CIs for IR progressively increased across tertiles of each parameter, and the TyG and modified TyG indices predicted IR significantly in the ROC curve analysis. Overall, modified TyG indices presented higher ORs and 95% CIs for predicting IR than the TyG index and lipid profiles. Among all indices, TyG-WHtR showed the strongest association with HOMA-IR, and TyG-BMI SDS was the most powerful predictor for IR in total subjects. In addition, TyG-WHtR was superior to TyG-WC for predicting IR in the present study.

IR plays an important role in type 2 diabetes, metabolic syndrome, and CVD [23]. Therefore, early detection of IR in people at risk for future CVD is important. Among the detection methods, TyG and modified TyG indices have been proposed as reliable markers in adults [16,24,25]. A population-based cross-sectional study suggested that the TyG index is useful for IR prediction in adults [26]. Another longitudinal study reported that the TyG index predicts type 2 diabetes in middle-aged and older adults [27]. However, elaborate studies that investigated the relationship between the TyG and modified TyG indices in youth are extremely limited.

Several studies have validated the relationship between the TyG index and IR. First, hypertriglyceridemia may increase hepatic glucose output with the increased transport of free fatty acids to the liver, making it one of the important risk factors for type 2 diabetes [28,29]. Research has shown that TG elevation can induce IR through the impairment of muscle glucose metabolism [30]. Second, insulin accelerates adipocyte TG stores by promoting TG synthesis and inhibiting lipolysis as well as promoting the maturation of adipocytes [28]. In addition, insulin stimulates lipoprotein lipase activity, thus increasing the uptake of fatty acids from circulating lipoproteins.

Obesity is strongly associated with IR [16]. Therefore, a combination of the TyG index and obesity parameters may predict IR better than the TyG index alone. Because BMI is a simple and widely used indicator of obesity and other metabolic risks, TyG-BMI can be a useful predictive marker [25]. Lim et al. [16] reported that TyG-BMI was superior to other modified TyG indices for predicting IR in adults. In children, obesity is defined as a BMI of ≥95th percentile of sex- and age-specific references; thus, BMI SDS should be used more commonly than BMI itself [31]. Therefore, we suggested a new parameter, TyG-BMI SDS, to explain obesity better than TyG-BMI in youth. It exhibited the largest AUC for the prediction of IR among all parameters in total subjects and females. 

Measures of central obesity, such as WC and WHtR, have been suggested as better indices than BMI because central obesity is closely associated with fat distribution but not BMI [32]. A meta-analysis revealed that measures of central obesity predict cardiovascular risk factors, including diabetes, hypertension, and dyslipidemia better than BMI [33]. A systematic review reported that combining BMI with measures of central obesity is superior to using BMI alone to assess the mortality risk of patients with coronary artery disease [34]. Among the measures of central obesity, WC does not directly reflect the difference in height, and age-dependent WC cutoffs are required [35]. However, WHtR has been reported to outperform WC and BMI in predicting metabolic syndrome and cardiovascular risks accounting for height, especially in Asians [36]. Overweight children with a higher WHtR are more likely to have higher cardiometabolic risk factors [32,37]. Thus, we analyzed both TyG-WHtR and TyG-WC and found TyG-WHtR to be superior to TyG-WC in predicting IR in youth. In contrast, TyG-WC was superior to TyG-WHtR in an adult study [16]. 

High-carbohydrate diet increases insulin level, which raises TG synthesis and glucose level [28,29,38]. Thus, response to dietary carbohydrate restriction might provide an operational definition of metabolic syndrome [38]. Carbohydrate restriction is considered as effective nutritional therapy for metabolic syndrome [39,40,41]. Thus, the ability of carbohydrate restriction and ketogenic diets to control the markers of the metabolic syndrome has been suggested [42,43]. Our study investigated association of the TyG and modified TyG indices with IR in the population with cross-sectional data. Further studies investigating improvement in the TyG and modified TyG indices after dietary carbohydrate restriction and ketogenic diet could make these parameters even more robust.

This study has some limitations. First, this was a cross-sectional study exclusive to Korean youth, thus limiting the generalizability of our findings. Second, associated factors, such as pubertal status, diet, and physical activity, were not considered. Third, lean and fat body mass were not considered in this study. Despite these limitations, the present study assessed the TyG and modified TyG indices as markers of IR across a large number of children and adolescents. In addition, we proposed a new parameter, TyG-BMI SDS, as one of the important predictors of IR in youth. 

## 5. Conclusions

This study indicates that the TyG and modified TyG indices could serve as valuable predictors of IR in youth. Moreover, combinations of obesity parameters with the TyG index—including the new parameter, TyG-BMI SDS—have better diagnostic accuracy than the TyG index. The TyG and modified TyG indices are simple and cost-effective markers of IR. Thus, these markers are useful for the assessment of cardiometabolic risk factors.

## Figures and Tables

**Table 1:** Baseline characteristics of participants according to sex.

	Total (n = 3728)	Male (n = 1756)	Female (n = 1972)	*p*
Age (years)	14.56 (0.06)	14.53 (0.07)	14.60 (0.09)	0.476
Height (cm)	161.79 (0.23)	165.53 (0.33)	157.52 (0.22)	<0.001
Height SDS	0.21 (0.02)	0.24 (0.03)	0.17 (0.03)	0.063
Weight (kg)	54.96 (0.29)	58.80 (0.42)	50.56 (033)	<0.001
Weight SDS	0.03 (0.03)	0.07 (0.04)	−0.02 (0.04)	0.056
BMI (kg/m^2^)	20.73 (0.07)	21.17 (0.10)	20.23 (0.11)	<0.001
BMI SDS	−0.09 (0.03)	−0.06 (0.04)	−0.13 (0.04)	0.225
WC (cm)	70.11 (0.21)	72.42 (0.29)	67.48 (0.27)	<0.001
WC > 90p	16.45% (0.75)	14.75% (0.98)	18.40% (1.18)	0.020
WHtR	0.43 (0.01)	0.44 (0.02)	0.43 (0.02)	<0.001
Glucose (mg/dL)	88.82 (0.19)	89.32 (0.25)	88.24 (0.20)	<0.001
Insulin (µIU/mL)	13.62 (0.18)	13.53 (0.25)	13.71 (0.23)	0.574
HOMA-IR	3.04 (0.05)	3.05 (0.08)	3.03 (0.06)	0.834
IR *	12.03% (0.68)	13.19% (0.90)	10.69% (0.93)	0.044
TC (mg/dL)	158.25 (0.58)	154.59 (0.81)	162.44 (0.74)	<0.001
LDL-C (mg/dL)	91.23 (0.50)	88.96 (0.69)	93.82 (0.65)	<0.001
Non-HDL-C (mg/dL)	108.65 (0.56)	106.35 (0.78)	111.28 (0.73)	<0.001
TG (mg/dL)	88.55 (1.16)	88.60 (1.62)	88.50 (1.50)	0.962
HDL-C (mg/dL)	49.61 (0.20)	48.25 (0.25)	51.16 (0.28)	<0.001
AIP	0.46 (0.01)	0.48 (0.02)	0.441 (0.02)	0.083
TyG index	8.14 (0.01)	8.14 (0.02)	8.14 (0.01)	0.650
TyG-BMI	169.17 (0.69)	172.89 (0.99)	164.91 (0.95)	<0.001
TyG-BMI SDS	−0.58 (0.22)	−0.277 (0.30)	−0.931 (0.32)	0.140
TyG-WC	571.93 (2.05)	591.16 (2.93)	549.94 (2.47)	<0.001
TyG-WHtR	3.54 (0.01)	3.57 (0.02)	3.49 (0.02)	0.003

Values are presented as mean (standard error), and categorical data as percentages (standard error). * IR was defined as the HOMA-IR of >95th percentile for each sex and age. SDS, standard deviation score; BMI, body mass index; WC, waist circumference; WHtR, waist-to-height ratio; HOMA-IR, homeostasis model assessment of insulin resistance; IR, insulin resistance; TC, total cholesterol; LDL-C, low-density lipoprotein cholesterol; HDL-C, high-density lipoprotein cholesterol; TG, triglycerides; AIP, atherogenic index of plasma; TyG, triglyceride glucose index.

**Table 2:** Odds ratio for insulin resistance according to tertiles of each parameter.

	Total		Male		Female	
	OR (95% Cl)	*p*	OR (95% Cl)	*p*	OR (95% Cl)	*p*
TC (mg/dL)						
T2	1.10 (0.82–1.49)	0.133	1.38 (0.90–2.10)	0.515	0.73 (0.46–1.14)	0.066
T3	1.75 (1.30–2.36)	<0.001	2.36 (1.61–3.47)	<0.001	1.09 (0.72–1.65)	0.175
LDL-C (mg/dL)						
T2	0.83 (0.62–1.11)	0.010	1.16 (0.79–1.71)	0.317	0.61 (0.39–0.93)	0.018
T3	1.34 (1.00–1.80)	0.003	1.88 (1.26–2.78)	0.001	0.94 (0.62–1.44)	0.329
Non-HDL-C (mg/dL)						
T2	1.02 (0.75–1.40)	0.004	1.12 (0.74–1.68)	0.005	0.73 (0.44–1.19)	0.027
T3	2.26 (1.66–3.08)	<0.001	3.28 (2.19–4.89)	<0.001	1.34 (0.89–2.14)	0.012
TG (mg/dL)						
T2	1.96 (1.35–2.84)	0.706	2.65 (1.63–4.30)	0.925	1.28 (0.76–2.14)	0.340
T3	4.25 (2.99–6.05)	<0.001	6.78 (4.23–10.88)	<0.001	2.45 (1.56–3.86)	<0.001
HDL-C (mg/dL)						
T2	0.61 (0.47–0.79)	0.544	0.63 (0.45–0.89)	0.764	0.38 (0.25–0.58)	0.004
T3	0.43 (0.32–0.59)	<0.001	0.44 (0.29–0.67)	0.003	0.45 (0.30–0.68)	0.114
AIP						
T2	1.82 (1.26–2.63)	0.565	2.66 (1.65–4.27)	0.693	1.27 (0.77–2.30)	0.385
T3	3.90 (2.78–5.46)	<0.001	6.14 (3.88–9.72)	<0.001	2.33 (1.48–3.66)	<0.001
TyG index						
T2	2.77 (1.84–4.16)	0.427	3.28 (1.92–5.56)	0.596	1.90 (1.09–3.30)	0.857
T3	6.06 (4.13–8.89)	<0.001	8.76 (5.228–14.667)	<0.001	3.34 (2.05–5.45)	<0.001
TyG-BMI						
T2	2.21 (1.35–3.62)	0.013	2.75 (1.32–5.71)	0.048	2.02 (1.08–3.80)	0.240
T3	11.43 (7.11–18.36)	<0.001	20.59 (10.45–40.56)	<0.001	7.17 (4.10–12.55)	<0.001
TyG-BMI SDS						
T2	3.53 (2.03–6.16)	0.831	3.438 (1.661–7.119)	0.330	3.580 (1.771–7.235)	0.430
T3	13.50 (7.84–23.25)	<0.001	19.05 (9.43–38.50)	<0.001	8.78 (4.58–16.83)	<0.001
TyG-WC						
T2	1.732 (1.109–2.707)	<0.001	2.476 (1.285–4.772)	0.023	1.92 (1.08–3.40)	0.286
T3	9.10 (5.92–13.99)	<0.001	17.44 (9.38–32.42)	<0.001	5.90 (3.47–10.05)	<0.001
TyG-WHtR						
T2	2.45 (1.42–4.22)	0.018	2.72 (1.27–5.79)	0.048	1.93 (1.03–3.63)	0.090
T3	14.66 (8.88–24.21)	<0.001	21.59 (11.15–41.80)	<0.001	8.25 (4.62–14.73)	<0.001

ORs and 95% CIs of tertiles 2–3 for each parameter were calculated and compared with those of tertile 1 as a reference. OR, odds ratio; CI, confidence interval; T, tertile; TC, total cholesterol; LDL-C, low-density lipoprotein cholesterol; HDL-C, high-density lipoprotein cholesterol; TG, triglycerides; AIP, atherogenic index of plasma; TyG, triglyceride glucose index; BMI, body mass index; SDS, standard deviation score; WC, waist circumference; WHtR, waist-to-height ratio.

**Table 3:** Cut-off values and areas under the receiver operating characteristic curves for each parameter for predicting insulin resistance.

	Cut-off	Sensitivity	Specificity	AUC	95% Cl	*p*
Total						
TyG index	8.261	66.449	65.555	0.723	(0.699–0.748)	<0.001
TyG-BMI	178.957	72.331	76.201	0.807	(0.785–0.829)	<0.001
TyG-BMI SDS	5.105	68.445	79.450	0.810	(0.788–0.831)	<0.001
TyG-WC	599.800	71.678	75.834	0.786	(0.763–0.810)	<0.001
TyG-WHtR	3.696	74.728	75.436	0.809	(0.788–0.831)	<0.001
Male						
TyG index	8.175	76.604	60.457	0.756	(0.726–0.786)	<0.001
TyG-BMI	184.058	79.245	77.680	0.842	(0.817–0.867)	<0.001
TyG-BMI SDS	5.137	76.190	78.274	0.838	(0.811–0.865)	<0.001
TyG-WC	600.847	83.019	70.299	0.820	(0.792–0.848)	<0.001
TyG-WHtR	3.719	81.132	75.220	0.842	(0.817–0.868)	<0.001
Female						
TyG index	8.260	64.433	63.508	0.680	(0.639–0.721)	<0.001
TyG-BMI	172.931	67.010	73.367	0.757	(0.720–0.794)	<0.001
TyG-BMI SDS	4.883	58.101	80.623	0.766	(0.730–0.802)	<0.001
TyG-WC	616.650	51.031	87.452	0.744	(0.705–0.783)	<0.001
TyG-WHtR	3.588	72.680	70.551	0.761	(0.725–0.797)	<0.001

AUC, area under the curve; TyG, triglyceride glucose index; BMI, body mass index; SDS, standard deviation score; WC, waist circumference; WHtR, waist-to-height ratio.

## Data Availability

The data presented in this study are available in this article.

## Appendix A

**Table A1:** Comparison of areas under receiver operating curves among each parameters for predicting insulin resistance.

	TyG index	TyG-BMI	TyG-BMI SDS	TyG-WC	TyG-WHtR
Total					
TyG index	reference	<0.001	<0.001	<0.001	<0.001
TyG-BMI	<0.001	reference	0.617	<0.001	0.739
TyG-BMI SDS	<0.001	0.617	reference	0.011	>0.999
TyG-WC	<0.001	<0.001	0.011	reference	<0.001
TyG-WHtR	<0.001	0.739	>0.999	<0.001	reference
Male					
TyG index	reference	<0.001	<0.001	<0.001	<0.001
TyG-BMI	<0.001	reference	0.505	<0.001	>0.999
TyG-BMI SDS	<0.001	0.505	reference	0.046	0.617
TyG-WC	<0.001	<0.001	0.046	reference	0.002
TyG-WHtR	<0.001	>0.999	0.617	0.002	reference
Female					
TyG index	reference	<0.001	<0.001	<0.001	<0.001
TyG-BMI	<0.001	reference	0.413	0.149	0.739
TyG-BMI SDS	<0.001	0.413	reference	0.143	0.721
TyG-WC	<0.001	0.149	0.143	reference	0.034
TyG-WHtR	<0.001	0.739	0.721	0.034	reference

Bootstrap method was used to perform pairwise comparisons between AUCs for the parameters. Values are presented as *p* values. TyG, triglyceride glucose index; BMI, body mass index; SDS, standard deviation score; WC, waist circumference; WHtR, waist-to-height ratio; AUC, area under the curve.
